# The 3′ Untranslated Region of the Rabies Virus Glycoprotein mRNA Specifically Interacts with Cellular PCBP2 Protein and Promotes Transcript Stability

**DOI:** 10.1371/journal.pone.0033561

**Published:** 2012-03-16

**Authors:** Saiprasad Palusa, Christina Ndaluka, Richard A. Bowen, Carol J. Wilusz, Jeffrey Wilusz

**Affiliations:** 1 Department of Microbiology, Immunology and Pathology, Colorado State University, Fort Collins, Colorado, United States of America; 2 Department of Biomedical Sciences, Colorado State University, Fort Collins, Colorado, United States of America; UMDNJ-New Jersey Medical School, United States of America

## Abstract

Viral polymerase entry and pausing at intergenic junctions is predicted to lead to a defined polarity in the levels of rhabdovirus gene expression. Interestingly, we observed that the rabies virus glycoprotein mRNA is differentially over-expressed based on this model relative to other transcripts during infection of 293T cells. During infection, the rabies virus glycoprotein mRNA also selectively interacts with the cellular poly(rC)-binding protein 2 (PCBP2), a factor known to influence mRNA stability. Reporter assays performed both in electroporated cells and in a cell-free RNA decay system indicate that the conserved portion of the 3′ UTR of the rabies virus glycoprotein mRNA contains an RNA stability element. PCBP2 specifically interacts with reporter transcripts containing this 72 base 3′ UTR sequence. Furthermore, the PCBP2 interaction is directly associated with the stability of reporter transcripts. Therefore, we conclude that PCBP2 specifically and selectively interacts with the rabies virus glycoprotein mRNA and that this interaction may contribute to the post-transcriptional regulation of glycoprotein expression.

## Introduction

Regulation of mRNA levels in eukaryotic cells is achieved by a combination of factors that influence either the synthesis or decay of the transcript [1], [2]. Many of the elements that regulate mRNA decay have been localized to the 3′ UTR of the targeted transcript [3], and regulation is mediated by the interaction of proteins or noncoding regulatory RNAs (miRNAs) with these elements [4], [5]. While extensive work has been done on the identification of promoter elements and transcriptional regulation of RNA virus gene expression, the post-transcriptional regulation of viral mRNAs at the level of transcript stability remains understudied [6], [7]. The goal of this study was to investigate a potential contribution of post-transcriptional regulation to the expression of rabies virus genes.

Rabies virus encodes five independent mRNAs which are transcribed from its single stranded, negative sense genomic RNA [6], [8]. The rabies virus RNA-dependent RNA polymerase enters exclusively at the 3′ end of the genome and sequentially transcribes individual mRNAs from each gene [6]. The polymerase pauses at each intergenic region and re-initiates synthesis at the downstream gene with about 70% efficiency on isolated nucleocapsids [8]. This ‘stop-start’ mode of transcription leads to relatively high levels of the 3′ terminal nucleocapsid (N) mRNA, but progressively less phosphoprotein (P), matrix (M), glycoprotein (G) and polymerase (L) transcripts [6], [8]. Since each of the five mRNAs contains an independent and unique 5′ and 3′ UTR segment, it is possible that message-specific post-transcriptional regulation of gene expression at the level of differential mRNA stability or translation could play a supporting role in determining the efficiency and coordination of rabies virus (and other negative sense RNA virus) infections.

The biology of a natural rabies virus infection is rather complex when compared to other rhabdoviral infections [9]. The virus must successfully replicate not only at the site of infection, but also in neuronal tissue as it moves towards the central nervous system. Since this process can take months, the virus must also be able to persist and avoid stimulating a neutralizing host immune response during this time. Thus careful regulation of surface glycoprotein expression may be particularly important for the successful progression of rabies virus infection. Regulated expression of the G protein has been clearly documented [10], [11] and variations in the non-coding regions of this gene that may have regulatory influence are common in rabies virus isolates [12]. However the minimal 3′ UTR that is conserved in all rabies virus isolates sequenced to date is a 72 base sequence typified by the laboratory-adapted ERA strain [13]. Therefore the rabies virus glycoprotein mRNA appears to be a particularly attractive candidate for additional investigations into post-transcriptional regulation of its expression.

In this study, we demonstrate that rabies virus G mRNA selectively and specifically associates with the cellular poly(rC)-binding protein 2 (PCBP2), a multi-functional RNA-binding protein that has been demonstrated to regulate mRNA stability and translation [14]–[18]. Interestingly, rabies virus G mRNA levels are elevated relative to other viral mRNAs, contrary to what would have been expected if stop-start transcriptional regulation were the sole determinant of viral transcript levels. A variety of complementary assays are used to demonstrate that an RNA stability element is present in the conserved portion of the 3′ UTR of rabies virus G mRNA. Furthermore, PCBP2 binding is directly associated with mRNA stability conferred by the rabies virus G 3′ UTR. Therefore PCBP2 appears to be a transcript-specific cellular factor that can influence rabies virus gene expression.

## Results

### The rabies virus glycoprotein mRNA selectively interacts with the cellular PCBP2 protein during infection

Cellular mRNAs possess a variety of regulatory elements that result in an extensive degree of post-transcriptional regulation of gene expression [3]. Proteins that interact with mRNAs often play a major role in eliciting this regulation. Many aspects of potential post-transcriptional control of individual rabies virus mRNAs remain under-explored. In order to begin to address this, we set out to determine unique aspects of specific rabies viral mRNPs.

**Figure 1:**
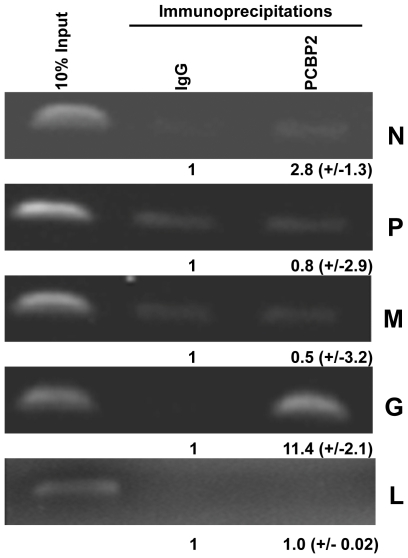
The cellular PCBP2 protein selectively interacts with the G mRNA during rabies virus infection. Rabies virus infected 293T cells were treated with formaldehyde to stabilize RNA-protein complexes and cell lysates were immunoprecipitated using either control IgG or a PCBP2-specific antisera. Co-precipitating rabies viral mRNAs were analyzed by RT-PCR after reversal of the formaldehyde cross links and products run on a 1% agarose gel and visualized with ethidium bromide. The 10% input lane represents total RNA present in the lysate prior to immunoprecipitation. The numbers below the lanes represent quantification and standard deviation from three independent experiments.

The interaction of individual rabies virus mRNAs with select cellular RNA binding proteins was pursued using a co-immunoprecipitation approach. Human embryonic kidney (293T) cells were infected with rabies virus and RNA-protein complexes were stabilized using formaldehyde prior to cell lysis. Protein-RNA complexes were immunoprecipitated under stringent conditions and associated RNAs were detected by PCR. While most of the RNA binding proteins tested gave negative or inconclusive results, we made an interesting observation with the cellular protein PCBP2. As seen in Figure 1, the rabies virus G mRNA was selectively associated with PCBP2. All four of the other rabies virus mRNAs did not co-immunoprecipitate with PCBP2 significantly above background. Therefore we conclude that the rabies virus G mRNA selectively associates with the cellular PCBP2 protein during infection.

### The rabies virus glycoprotein mRNA is selectively over-expressed during infection

**Figure 2:**
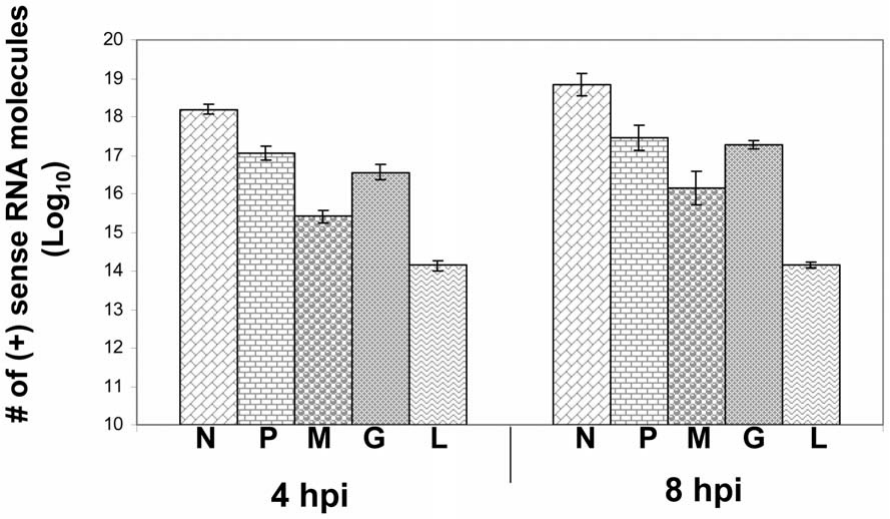
The rabies virus G mRNA is overexpressed relative to other viral transcripts. Total RNA was isolated from rabies virus infected 293T cells at the times indicated post infection. qRT-PCR assays were performed for each of the viral mRNAs and Ct values converted to numbers of RNA molecules per µg of total cellular RNA using a standard curve that was developed for each set of primers.

PCBP2 is a multifunctional post-transcriptional regulator of gene expression that can influence both mRNA stability and translation [14]–[18]. In order to investigate its possible impact on glycoprotein mRNA expression, we first assessed the relative expression of the G mRNA by qRT-PCR. Primers for all five rabies virus mRNAs were standardized and used against a dilution series of linearized cDNA plasmids in order to allow an accurate conversion of Ct values to numbers of mRNA molecules. Rabies virus infections were performed in 293T cells and total RNA was isolated at 4 or 8 hours post infection. As seen in Figure 2, most of the rabies virus mRNAs showed the expected polarity of gene expression based on their genomic position relative to the 3′ end according to the stop-start model of transcriptional regulation [8]. At both 4 and 8 hpi, the G mRNA, on the other hand, was clearly an exception to this model. While models of transcriptional regulation would predict that the G mRNA would be ∼70% or less of the level of the mRNA generated from the upstream M gene [19], G mRNAs were significantly higher, approaching the level of the P mRNA. Since the amount of antigenomic RNA detected by each primer set should be roughly consistent, the higher levels of RNA molecules detected by the G mRNA probe must reflect an unexpected over-expression of this specific mRNA.

Thus it appears that the expression levels of the G mRNA cannot be solely explained by transcriptional regulation and the ‘stop-start’ action of rabies virus polymerase on the viral genomic RNA.

### The conserved portion of the 3′ UTR of the rabies virus G mRNA contains RNA stability determinants

Cellular mRNA levels are determined by a combination of the relative rates of transcription and degradation of the transcript [1]–[4]. Differential mRNA stability can, in fact, play a major role in determining transcript levels in cells [1], [2]. Therefore we hypothesized that differential mRNA stability may be making a significant contribution to the surprisingly high relative levels of the rabies virus G mRNA compared to what would be expected from its synthetic rate alone.

Our efforts to directly assay the stability of the G mRNA in the context of a rabies virus infected cell were thwarted by two technical hurdles. First, we do not have access to a rabies virus variant that contains a temperature sensitive allele in the polymerase complex that would allow us to shut off viral transcription and accurately measure viral mRNA half lives. Second, we could not successfully apply pulse-chase labeling technologies using 4-thio uridine [2], [20], [21] due to the fact that changing numbers of transcription templates caused by extensive viral replication confounds the analysis of the relative contribution of mRNA decay. Therefore we turned to two independent approaches using reporter constructs that have been successfully applied in the past to study RNA decay of cytoplasmic viruses [22].

**Figure 3:**
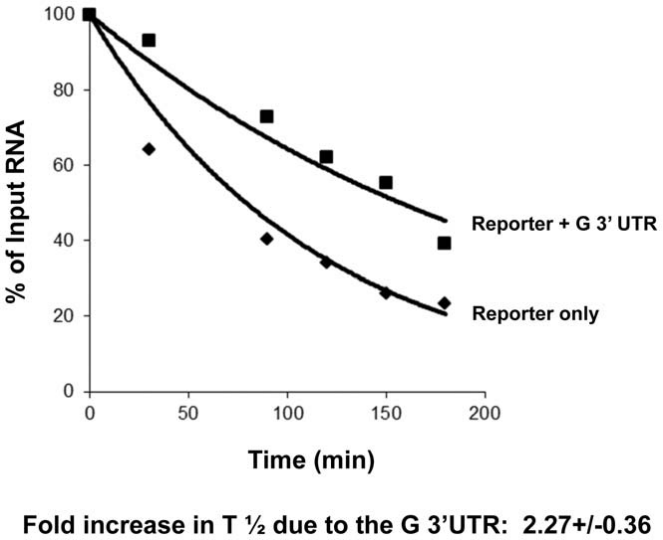
Electroporated reporter RNAs can be stabilized by a conserved segment of the rabies virus G 3′UTR. A radiolabeled, capped and polyadenylated reporter RNA derived from pGem-A60 (reporter only), or a variant containing a 72 base segment from the 3′ UTR of the rabies virus G mRNA (reporter+G 3′ UTR), were electroporated into BHK cells. Total RNA was isolated from cells at the times indicated, analyzed on a 5% polyacrylamide gel containing urea, and visualized by phosphorimaging. A graphical representation of the results obtained independent representative experiment is shown. The standard deviation shown in the calculation of half-life differences was determined from 3 independent experiments.

First, we inserted the conserved 72 base 3′ UTR segment of the G mRNA that is present in all rabies virus isolates into a reporter construct and electroporated radiolabeled capped and polyadenylated transcripts into the cytoplasm of BHK cells. We have previously validated this electroporation assay by successfully using it to reveal differential regulation of deadenylation and exonucleolytic decay [22]. As seen in Figure 3, while the reporter-only control construct decayed fairly rapidly, the reporter containing the segment from the rabies virus G mRNA was significantly stabilized. RNA half-life calculations indicated that the inclusion of the G mRNA 3′ UTR segment resulted in a ∼two-fold stabilization of the transcript relative to control RNAs.

**Figure 4:**
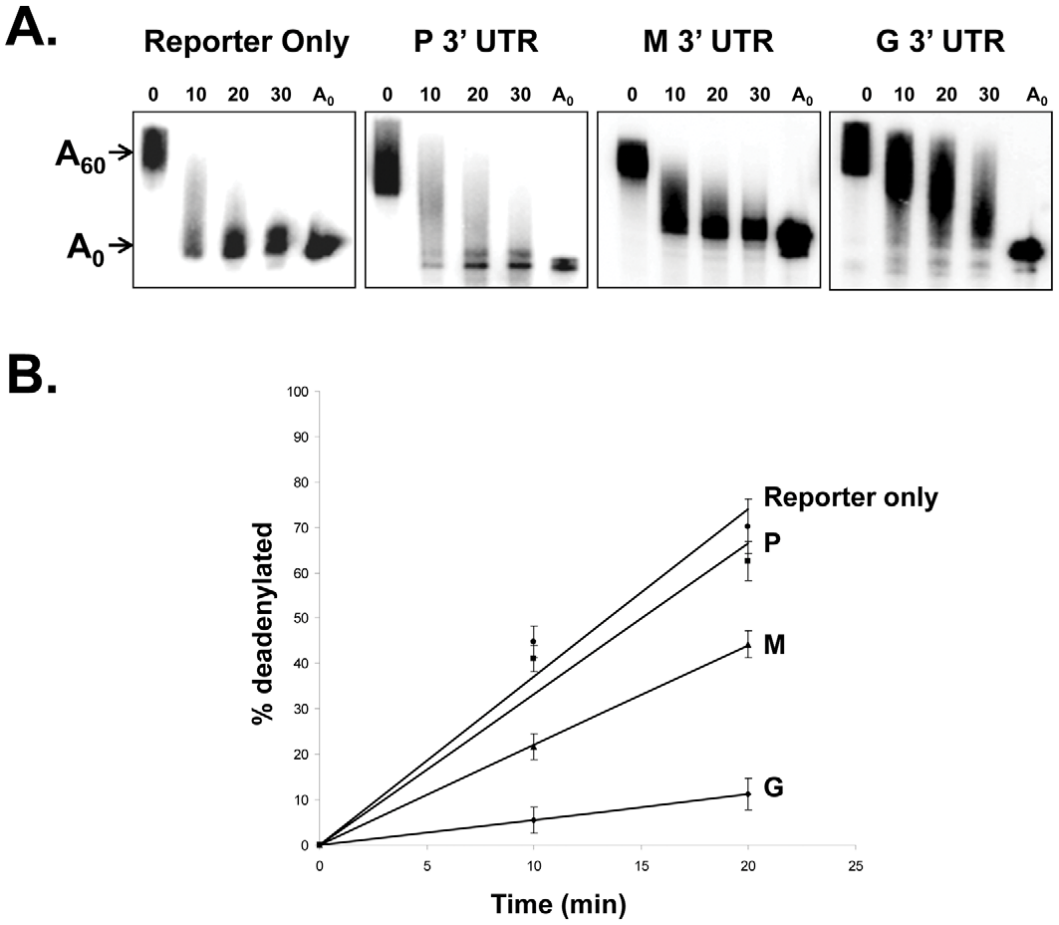
The 3′ UTR of the rabies virus G mRNA represses deadenylation of a reporter RNA in a cell free deadenylation/decay assay. Panel A: Radiolabeled capped and polyadenylated RNAs containing either reporter only (Gem A60) or the reporter plus the 3′ UTR of the rabies virus P, (P 3′ UTR), M, (M 3′ UTR) or G (G 3′ UTR) mRNAs were incubated with HeLa cytoplasmic extract in a cell-free deadenylation/decay assay. Aliquots were taken at the times indicated and reaction products were analyzed on a 5% polyacrylamide gel containing urea. The position of the polyadenylated (A_60_) input RNA is indicated in the ‘0’ lane and a deadenylated marker RNA is run in the ‘A_0_’ lane. Panel B: Graphical representation of three independent experiments as described in Panel A. The error bars represent standard deviations.

To confirm this observation using an independent assay, we prepared radiolabeled capped and polyadenylated reporter constructs containing 3′ UTR segments from the P, M and G genes of rabies virus and incubated them in a cell-free mRNA deadenylation/decay system that we have previously reported using HeLa cytoplasmic extracts [23]. This cell free RNA decay assay faithfully reproduces a variety of aspects of regulated RNA stability that is observed in living cells [24]–[26]. As seen in Figure 4, reporter constructs containing the conserved portion of the G mRNA were deadenylated very slowly in the these assays, while the reporter-only construct or versions containing the M or P 3′ UTRs were rapidly deadenylated. The inclusion of the 72 base G mRNA 3′ UTR segment resulted in an approximate four-fold stabilization of RNAs in this assay while the P and M 3′ UTR segments had no significant effect. Therefore, collectively based on the results obtained from the electroporation and cell-free assay studies, we conclude that the conserved portion of the 3′ UTR of the G mRNA does indeed contain RNA stabilizing determinants.

### The interaction of PCBP2 with the conserved portion of the rabies virus G mRNA 3′ UTR is associated with increased RNA stability

Finally, we wished to directly connect PCBP2 interactions with RNA stability mediated by the conserved portion of the 3′ UTR of the rabies virus G mRNA. Since knockdown of PCBP2 and its highly conserved (∼90% identical) isoform PCBP1 can cause cell cycle arrest in cells [27], we turned to our cell free assays to avoid any potential indirect effects due to changes in cell growth.

**Figure 5:**
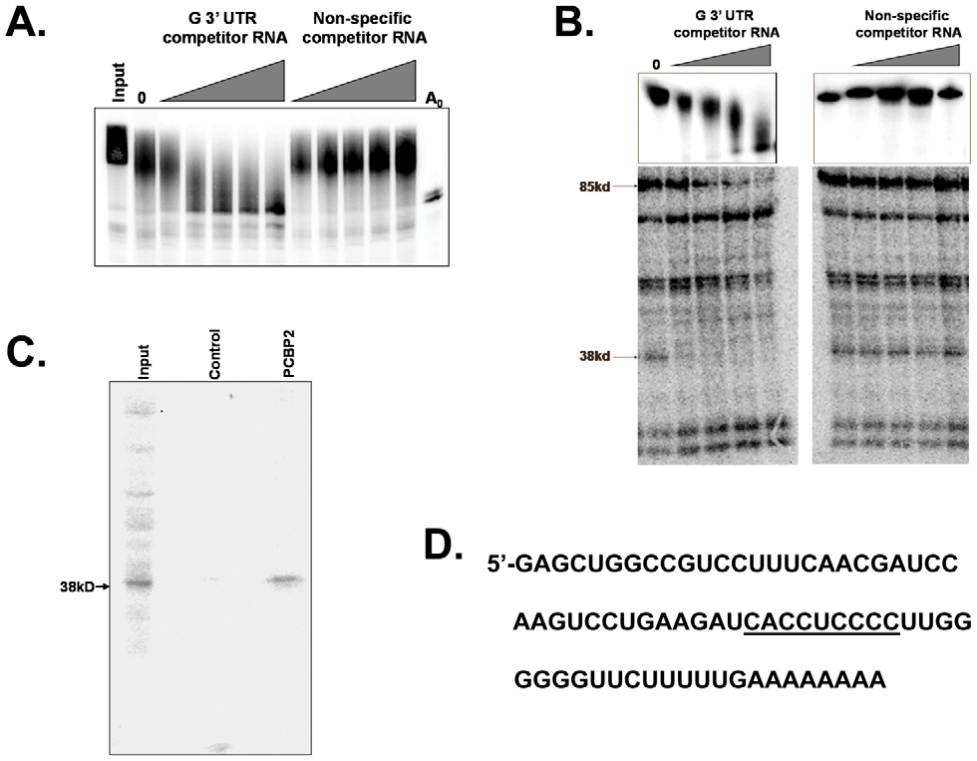
A titratable *trans*-acting factor is required for the repression of deadenylation mediated by the 3′ UTR of the rabies virus G mRNA. Panel A. Radiolabeled, capped and polyadenylated reporter RNA containing the 3′ UTR of the rabies virus G mRNA was incubated either in the absence (lane 0) or the presence of the indicated amount and type of competitor RNA for 10 minutes in the cell-free RNA deadenylation/decay assay using HeLa cytoplasmic extract. RNA products were isolated and analyzed on a 5% polyacrylamide gel containing urea. Panel B. Top: Competition assays using a capped and polyadenylated radiolabeled reporter RNA containing the 3′ UTR of the rabies virus G mRNA were performed as described in Panel A. Bottom: In parallel reactions, protein-RNA interactions with the radiolabeled RNA substrate in the presence of the indicated types and relative amounts of competitor RNA in the cell-free RNA deadenylation/decay system were assessed. EDTA was added to these reactions to block RNA degradation and afford a valid comparison between lanes. Samples were treated with short wave UV light, incubated with RNase One, and proteins covalently attached to short radioactive RNA oligomers were resolved on a 10% polyacrylamide gel containing SDS. Results were visualized by phosphorimaging. Panel C. Radiolabeled reporter RNA containing the 3′ UTR of the rabies virus G mRNA was incubated with HeLa cytoplasmic extract in the cell-free RNA deadenylation/decay system, UV cross linked and processed as described for Panel B, and analyzed by 10% SDS-PAGE either directly (input lane) or after immunoprecipitation with non-specific IgG (control lane) or with PCBP2-specific antibodies (PCBP2) lane. The positions of the 38 kDa and 85 kDa proteins are indicated on the left of the protein gels. Panel D: The sequence of the 72 base conserved region of the rabies virus G mRNA that was used in these experiments. A C-rich region, representing a possible PCBP2 binding site, is shown.

In order to ascertain whether a titratable *trans*-acting factor was involved in RNA stability mediated by the 3′ UTR of the rabies virus G mRNA, we first performed competition assays using either a specific rabies virus G 3′ UTR transcript or a non-specific control RNA to assess whether deadenylation of reporter RNAs containing the conserved portion of the G 3′UTR could be activated. As seen in Figure 5A, increasing amounts of a G 3′ UTR competitor effectively activated deadenylation/decay of the reporter RNA. Equal amounts of a control competitor RNA, however, had no effect. Therefore we conclude that a titratable *trans*-acting factor, presumably a protein, is required for RNA stabilization mediated by the conserved 72 base portion of the rabies virus G mRNA.

In order to identify this titratable factor, we performed competition assays as described for Figure 5A but also included an analysis of protein-RNA interactions by UV cross linking in parallel with the analysis of deadenylation/decay of the reporter. As seen in Figure 5B, the non-specific competitor RNA failed to activate deadenylation/decay or appreciably compete for any of the proteins that interacted with the radiolabeled capped and polyadenylated reporter RNA containing the rabies virus G 3′ UTR. However a specific competitor RNA that contained the conserved 72 base G mRNA 3′ UTR sequence both activated deadenylation and competed for the interaction of two proteins (85 kDa and 38 kDa) with the reporter RNA containing the rabies virus G 3′ UTR. Thus these two proteins represent attractive candidates for factors that regulate RNA stability by binding to this 72 base sequence. Interestingly, PCBP2 protein isolated from HeLa cells migrates at 38 kDa. As seen in Figure 5C, immunoprecipitation of proteins UV cross linked to a radiolabeled reporter RNA containing the conserved portion of the rabies virus G 3′ UTR confirms that the 38 kDa protein is indeed PCBP2. Thus the interaction between PCBP2 and RNAs containing the conserved portion of the rabies virus G 3′ UTR (which contains a C-rich region underlined in Fig. 5D) is directly associated with transcript stability.

**Figure 6:**
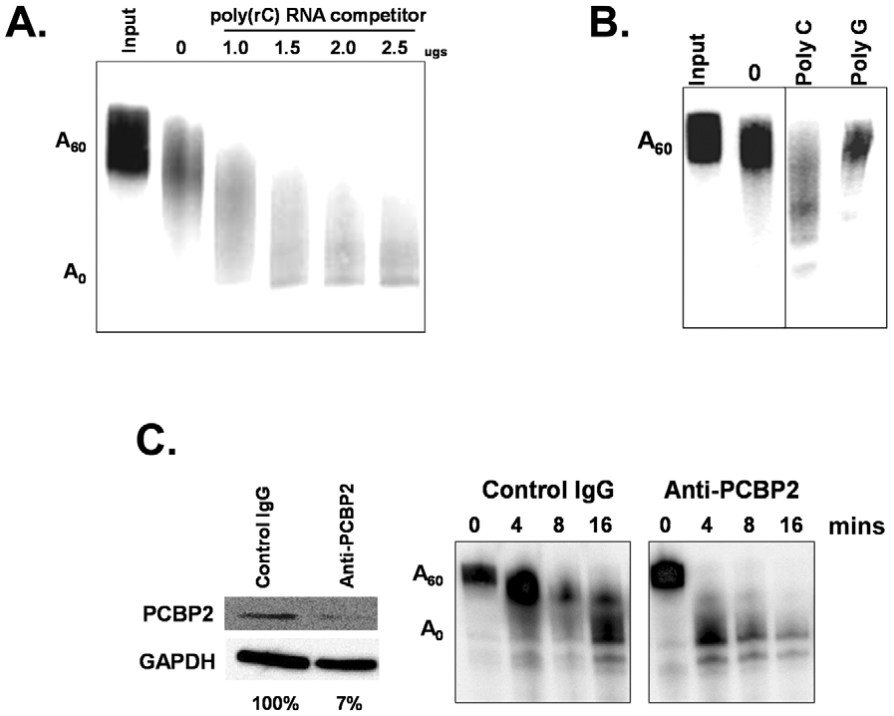
The protein responsible for stabilizing reporter RNAs containing the 3′ UTR of the rabies virus G mRNA is a poly(C) binding protein. Panel A. Radiolabeled, capped and polyadenylated reporter RNA containing the 3′ UTR of the rabies virus G mRNA was incubated in the cell-free RNA deadenylation/decay system using HeLa cytoplasmic extract for 10 minutes in the presence of the indicated amount of poly(C) competitor RNA. Reaction products were analyzed on a 5% polyacrylamide gel containing urea. The positions of the input polyadenylated (A_60_) and deadenylated product (A_0_) are indicated at the left of the gel. The lane marked input indicates the RNA in a reaction that was set up but not incubated. Panel B: Same as panel A. 2.5 µgs of the indicated competitor RNA were added in the lanes marked poly C and poly G. The lane marked ‘0’ indicates a control deadenylation reaction to which no competitor RNA was added. Reactions were incubated for 10 minutes. Panel C: HeLa cytoplasmic extracts were immunodepleted using the indicated antisera. In the left panel, the extent of immunodepletion was examined by western blot using the indicated antisera. GAPDH was used as a normalization control. In the right panel, extracts immunodepleted using either control IgG or PCBP2-specific antibodies were used in our cell-free deadenylation assays with a reporter RNA containing the 3′ UTR of the G mRNA. Samples were taken at the times indicated and analyzed on a 5% polyacrylamide gel containing urea. The positions of the input polyadenylated (A_60_) and deadenylated product (A_0_) are indicated at the left of the RNA gel.

Finally, if PCBP2 is indeed the factor required for the high relative stability of the rabies virus G reporter constructs in these assays, the addition of poly(C), but not other homopolymers, should titrate away the protein [28] and cause a dramatic increase in the deadenylation/decay rate of the reporter transcript. As seen in Figure 6A, this is precisely the case. The addition of poly(C) causes a clear increase in the deadenylation/decay rate of a reporter RNA containing the rabies virus G 3′ UTR. The addition of similar amounts of poly(G), however, had no effect on transcript stability (Fig. 6B). Importantly, immunodepletion of PCPB2 to 7% of the level present in control IgG treated extracts results in increased deadenylation of a reporter RNA containing the rabies virus G 3′ UTR in our cell-free RNA stability assays (Fig. 6C). Immunodepletion using non-specific control IgG had no effect in parallel experiments. Therefore we conclude that PCBP2 can stabilize RNAs containing a conserved portion of the 3′ UTR of the rabies virus G mRNA.

## Discussion

In this study we demonstrate that the rabies virus G mRNA is differentially over-expressed during infection compared to other viral transcripts and that it selectively interacts with the cellular PCBP2 protein. Using a set of complementary assays, the conserved portion of the 3′ UTR of the rabies virus G mRNA was shown to possess an RNA stabilizing element whose activity required a titratable *trans*-acting factor. The PCBP2 protein, a previously characterized RNA stability factor [14], [15], was shown to interact with this 3′ UTR region and this interaction could be directly associated with increased transcript stability. Therefore we suggest that PCBP2 is a cellular protein that may be usurped by rabies virus during infection to afford differential regulation of expression of its glycoprotein mRNA.

Previous investigations of rabies virus gene expression have focused largely on transcriptional regulation and applications of the stop-start model of polymerase entry/re-initiation [8], [19]. Since negative sense RNA viruses like rabies virus generate multiple independent transcripts during infection that contain largely distinct 5′ and 3′ UTRs, we propose that differential mRNA stability may also contribute to the levels and regulation of rabies virus gene expression in certain cells/tissues. In this manner, rabies virus would attain another important means of regulating relative mRNA levels that would give the virus additional versatility in its gene expression during the variety of biological scenarios it encounters during natural infections. This versatility provided by differential mRNA stability is extremely important for the regulation of gene expression of cellular mRNAs, and there is no *a priori* reason why negative sense viruses that infect eukaryotic cells might not take advantage of this avenue as well.

PCBP2 has been previously shown to play a major role in regulating the stability of a variety of cellular mRNAs, most notably, α-globin [14]. PCBP2 has also been demonstrated to play a role in translational regulation [16]–[17] as well as P-body and stress granule trafficking [29]. Therefore PCBP2 appears to be a coordinator/regulator of the major cytoplasmic processes involved in gene expression of select mRNAs. Interestingly, a variety of other viruses use PCBP2 protein for one or more of these functions. The 5′ NCR of a variety of Picornavirus (e.g. poliovirus) mRNAs interact with PCBP2 to assist with their recruitment of the translation machinery to the viral IRES element as well as increase the overall replication of the virus [30]–[32]. Experiments to investigate the role of PCBP2 in rabies virus translation are planned for future studies. PCBP2 is also cleaved by viral-encoded proteases during a poliovirus infection, and this cleavage may help regulate the relative amounts of viral translation and replication [33]. Hepatitis C virus also commandeers the PCBP2 protein to assist with both its IRES-mediated translation activity as well as replication [34]. One interesting recent suggestion is that PCBP2, via its ability to oligomerize, might assist in the circularization of HCV 5′ and 3′ ends [35]. This circularization could assist the virus in a variety of ways, including perhaps increasing RNA stability by sequestering the ends of non-polyadenylated viral RNA that would be potential substrates for cellular exonucleases involved in RNA decay. Finally, there are reports that PCBP2 expression is stimulated during viral infection [36]. In addition to increasing the pool of PCBP2 available to the virus, PCBP2 also serves as a negative regulator of MAVS and the interferon response [36]. Thus interactions with PCBP2 appear to be rather common among RNA viruses and play important roles in virus biology and virus-host interactions.

While most interactions between viral transcripts and PCBP2 appear to be beneficial for the virus, this does not always appear to be the case. Like several RNA-binding proteins defined to date, PCBP2 may play a positive or negative role in gene expression depending on the context of its interactions [37]. Curiously, a recent report identified PCBP2 (and to a lesser extent PCBP1) as negative regulators of transcription of vesicular stomatitis virus (VSV) [38]. However the underlying mechanism for this down-regulation of VSV gene expression by PCBP2 is not clear. VSV is the prototypic member of the *Rhabdoviridae* family which contains rabies virus and other lyssaviruses. Thus, based on this recent report [38], the interactions between PCBP2 and the rabies virus G mRNA and the resulting RNA stabilization may not be generalizable to other negative sense viruses. This is perhaps not surprising given the novel biology of rabies virus infections compared to other rhabdoviruses as well as the extensive regulation of rabies virus glycoprotein expression via transcription and translation that has been previously documented [9], [10].

Whether or not PCBP2 requires other factors to modulate the stability of transcripts containing the rabies virus 3′ UTR remains to be investigated. Since SRp20 co-localizes with PCBP2 and assists in ribosome recruitment during poliovirus infections [39], the possibility of PCBP2 co-factors for mediating viral RNA stability is plausible. As seen in Figure 5, the interaction of an additional 85 kDa protein can be correlated with reporter RNA stability. We are currently attempting to identify this factor using an RNA affinity purification approach in conjunction with mass spectrometry. The identity of this factor, along with investigations into its relationship with PCBP2, may provide additional insights into the underlying role of PCBP2 in the glycoprotein expression during rabies virus infection.

In summary, this study has identified a novel host protein interaction with a specific rabies viral mRNA. While the ability of many positive-sense RNA viruses to produce transcripts with distinct 5′ and 3′ UTRs is rather limited, the expression strategy of negative sense RNA viruses to produce individual distinct mRNAs that interact with distinct sets of RNA binding proteins affords a potential level of regulatory control during infection that has not been extensively investigated to date. Thus additional investigations using RIP, HITS- or PAR-CLIP [40] or other technologies designed to assess protein-RNA interactions with specific viral transcripts during infection may yield novel insights into mechanisms that fine tune viral gene expression or allow viruses to adapt to different cellular environments.

## Materials and Methods

### Cell Culture and Rabies Virus Infections

Human 293T (ATCC CRL-11268) cells were cultured in DMEM, 10% fetal bovine serum (FBS), non-essential amino acids, L-glutamine, and penicillin/streptomycin in 5% CO_2_ at 37°C. BHK-21 (Baby hamster kidney, ATCC CCL-10) cells were grown at 37°C in 5% CO_2_ in complete medium [HyQ MEM/EBSS medium (HyClone) supplemented with 10% FBS, and 1% of each of the following: non-essential amino acids, L-glutamine (HyClone), and penicillin/streptomycin (HyClone)]. Cells were infected with rabies virus strain CVS-11 at a multiplicity of infection of 10 PFU per cell and harvested using TRIzol at the times indicated for RNA analysis.

### RNAs and In Vitro Transcription

Internally radiolabeled, capped RNAs were prepared by *in vitro* transcription from linearized plasmid DNA templates and gel purified as previously described [41]. Reporter RNA constructs were generated from pGem-A60 which contains a 60-base adenylate stretch followed immediately by an Nsi I restriction site inserted downstream of the Hind III site of the polylinker region of pGem-4. Cleavage of the plasmid with Nsi I and transcription using SP6 RNA polymerase generated a reporter RNA that contains a 60-base adenylate tract precisely at its 3′ end. Templates for generating reporter constructs containing rabies virus mRNA 3′ UTR sequences were obtained by inserting the following oligonucleotides and the appropriate complementary strand representing rabies virus mRNA 3′ UTR fragments between the BamH1 and Hind III sites of pGem-A60: L: ′5-GATCCTGTATTTTGAAAAAAACA; G: 5′- GATCCGAGCTGGCCGTCCTTTCAACGATCCAAGTCCTGAAGATCACCTCCCCTT GGGGGGTTCTTTTTGAAAAAAAA; P: 5′-5′GATCCCGAACCTCTCCACTCAGTCCCTCTAGACAATAAAGTCCGAGATGTCCTAAAGTCAACATGAAAAAAA; M (three fragment ligation): 5′- GATCCTCAGATTATATCCCGCAAATTTATCACTTGTTTACCTCTGGAGGAG AGAAACATATGGGCTCAACTCCAACCCTTGGGGGCAATATAACAAA and 5′-AAAACATGTTATGGTGCCATTAAACCGCTGCATTTCATCAAAGTCAAGTTAATTACCTTTACATTTTGATCCTCTTGGATGTGAAAAAAA. Transcription templates were generated by digestion with Hind III or Nsi I for the nonadenylated and adenylated transcripts, respectively.

### Analysis of Formaldehyde-Stabilized RNA-Protein Interactions

293T cells were infected with rabies virus as described above. Cells were released using trypsin, washed with phosphate-buffered saline (PBS), resuspended in 1% formaldehyde solution in PBS, and incubated for 10 minutes at room temperature. The reaction was quenched using 0.25 M glycine. Cell pellets were washed with PBS and resuspended in RIPA buffer [50 mM Tris-HCl (pH 7.5), 1% (v/v) NP-40, 0.5% (w/v) sodium deoxycholate, 0.05% (w/v) SDS, 1 mM EDTA, and 150 mM NaCl]. Cells were disrupted by sonication on ice, and insoluble materials were removed through centrifugation. Supernatant received anti-PCBP2 (MBL, International Corporation, Woburn, MA), or control (IgG) antibodies and were incubated at 4°C for 1 hr. Antibody-bound complexes were recovered using protein A Sepharose beads after 5 washes with RIPA buffer containing 1 M urea. Formaldehyde crosslinks were reversed by heating at 70°C for 45 min. RNA was isolated using TRIzol and analyzed by qRT-PCR to assess rabies virus mRNAs.

### Measurement of rabies virus mRNA levels during infection

At designated time points following rabies virus infection, cells were collected in TRIzol. RNA concentration, purity and quality were determined using a Bioanalyzer (Agilent). 1 µg of DNase-treated RNA was taken to prepare cDNA using reverse primers for specific rabies virus genes. These cDNA preparations were analyzed in Real-Time PCR assays using SYBR Green (Bio-Rad, Hercules, California). Rabies virus gene-specific qPCR primers were as follows: N: 5′- TATTGCTGCATGTGCTCCTC and 5′-TCATCTGCCAGTGCTACGTC; P: 5′- ATGGTCGGCTACCAATGAAG and 5′- ACGGGTCACACCTGGTACAT; M: 5′- ATTGGACTGGCTTTGTCAGG and 5′- ATTTGCAATCCGACGAACTC; G: 5′- CCTGGGTTTGGAAAAGCATA and 5′- CATTGCCGTCAGGTCCTAAT; L: 5′- CTGCTGATCGTGACAAAGGA and 5′- GACTTGATCCCCAGCAATGT.

The specificity of the primers was verified by analysis of qPCR melt curves and the predicted RT-PCR product size was confirmed by agarose gel electrophoresis of the PCR amplicon (data not shown). Primer titration and dissociation experiments were performed so that no primer dimers or false amplicons interfered with the result.

Absolute quantification of rabies virus mRNAs was approached by normalizing RT-PCR reaction with DNA standards. Plasmids containing cDNA of individual rabies virus mRNAs were linearized with Pst I and quantified by optical absorbance using a Nanodrop spectrophotometer. These DNAs were used in a dilution series in qPCR analyses to create standard curves using the Bio-Rad MyiQ iCycler and accompanying software. Total molecules of each rabies virus mRNA were calculated from standard curves based on Ct values that were obtained. Each sample had three replicates and infections/analyses were performed four times independently to generate the reported values.

### RNA Electroporation and Analysis of RNA Stability

BHK-21 cells were washed with 1× PBS, detached from plates using diluted trypsin and resuspended in PBS. Uniformly labeled, capped and polyadenylated RNA (1–10×10^6^ cpm) was electroporated into the cells in a 2-mm gap cuvette using an Electro Cell Manipulator 630B apparatus charged at 400 V, 800 Ω and 75 µF. After discharge, the cells were washed with PBS, resuspended in complete medium, plated and incubated at 37°C. At the time points indicated, cells were harvested, washed with PBS and total RNA was extracted using TRIzol. The RNAs were resolved on a 5% polyacrylamide gel containing 7 M urea and analyzed by phosphorimaging. RNA quantification was determined using Bio-Rad Quantity One software. The half-lives shown are averages of three separate electroporation experiments.

### Cell-Free RNA Deadenylation/Decay Assays

Hela cytoplasmic S100 extracts were prepared as described previously and used in cell-free deadenylation assays using a system previously developed in our laboratory [23]. Briefly, a typical reaction mixture contained 100,000 cpm (10 to 50 fmoles) of internally labeled RNA, 2.4% polyvinyl alcohol (PVA), 17.5 mM phosphocreatine, 0.7 mM ATP, 25 ng poly (A), 20 units of RNase inhibitor and 60% (vol/vol) S100 extract. Reaction mixtures were incubated at 37°C for the indicated times. Reaction products were recovered by phenol-chloroform extraction and ethanol precipitation, separated on a 5% polyacrylamide gel containing 7 M urea and RNA was analyzed by phosphorimaging. Results were quantified by assessing the relative level of fully deadenylated RNA versus the total RNA in the sample using Quantity One software. All experiments were repeated at least three times to generate numbers for statistical analysis.

To immunodeplete extracts, HeLa cytoplasmic extracts were treated with either control IgG or PCBP2-specific antisera (MBL, Woburn MA) for 1 hour at 4°C and cleared using protein A Sepharose beads prior to use in deadenylation assays as described above.

### UV cross-linking

UV cross-linking assays were performed as previously described [41] with some minor modifications. Radiolabeled RNAs were incubated in S100 extracts as described above for deadenylation assays with the addition of 5 mM EDTA to inhibit RNA decay. After incubation at 37° for 10 minutes, proteins were cross-linked to the radiolabeled RNA substrates using 254-nm UV light (1800J) in a UV Stratalinker 2400 (Stratagene). Reaction mixtures were treated with RNase A/RNase One for 15 minutes at 37°C and proteins cross-linked to short radioactive RNA oligomers were separated on a 10% SDS-PAGE gel and visualized by phosphorimaging. For UV cross-linking and immunoprecipitation assays samples were processed as described above. Following the RNase digestion step, NET2 buffer [50 mM Tris-HCl (pH 7.6), 150 mM NaCl, 0.01% (v/v) NP-40] was added and reactions were clarified of insoluble materials by centrifugation. Samples incubated with either control IgG or PCBP2-specific antibodies (MBL International Corporation, Woburn, MA) for 1 hr at 4°C. Formalin-fixed protein A-positive *Staphylococcus aureus* cells (Pansorbin) were then added and incubated at 4°C for 20 minutes. Antibody-antigen complexes were washed 5 times with NET2 buffer, and immunoprecipitated proteins were analyzed by 10% SDS-PAGE and visualized by phosphorimaging.
